# Benign convulsions with mild gastroenteritis in children: An emerging acute symptomatic seizures

**DOI:** 10.1002/pdi3.59

**Published:** 2024-05-20

**Authors:** Duan Wang, Lijuan Peng, Tingsong Li, Nong Xiao

**Affiliations:** ^1^ Department of Rehabilitation Children's Hospital of Chongqing Medical University (CHCMU) Chongqing China; ^2^ National Clinical Research Center for Child Health and Disorders Chongqing China; ^3^ Ministry of Education Key Laboratory of Child Development and Disorders Chongqing China; ^4^ China International Science and Technology Cooperation Base of Child Development and Critical Disorders Chongqing China; ^5^ Chongqing Key Laboratory of Pediatrics Chongqing China

**Keywords:** acute symptomatic seizures, children, convulsions, gastroenteritis, infant, self‐limited infantile epilepsy

## Abstract

Benign convulsions with mild gastroenteritis (CwG) characterized by afebrile seizures that occurred in the acute period of mild gastroenteritis often occur in infancy and toddlers. Until now, it has not been well acknowledged and thereby classified by the International League against Epilepsy (ILAE) as epilepsy syndrome or acute symptomatic seizures (ASS). Thus far, accumulating data suggest that CwG could fit all the mandatory criteria of ASS from the aspects of pathogenesis, clinical manifestations, and outcomes, rather than epilepsy in spite of the rare cases developing epilepsy over time. This review provides a comprehensive picture of this entity aiming to facilitate the pediatricians, particularly for general practitioners, to better recognize this unique entity and, ultimately, to minimize unnecessary evaluation and treatment.

## 1 INTRODUCTION

Since benign convulsions with mild gastroenteritis (CwG) was first reported by Morooak in 1982,^1^ a number of articles involving hundreds of cases were published that focused on the clinical features, Electroencephalogram (EEG) findings, and prognosis.^2^, ^3^, ^4^ In 1995, Komori proposed the definition and characteristics of CwG, which are as follows^5^: (1) previously healthy children without a history of convulsions or growth retardation between 6 months and 3 years of age; (2) patients in whom afebrile convulsions occurred during the acute phase of gastroenteritis; (3) patients with mild dehydration (<5% of body weight); (4) patients with normal laboratory findings, including those for cerebrospinal fluid and serum electrolyte and glucose tests; and (5) exclusion to patients diagnosed with febrile seizure, encephalitis, encephalopathy, and other neurological disorders.

To date, CwG has not been formally recognized as epilepsy syndrome or acute syndromic seizures by the International League Against Epilepsy (ILAE) because of the afebrile feature. Considering the common underlying genetic predisposition,^6^, ^7^ it has been proposed that CwG could likely be classified as benign infantile seizures (BIS).^8^, ^9^, ^10^ However, mounting evidences revealed that CwG is more likely to be considered as situation‐related seizure rather than epilepsy, from the aspects of age‐dependent profile, low risk of recurrence, and overall favorable prognosis.^11^, ^12^, ^13^, ^14^, ^15^ In this review, we discussed the published data and evidences that favored the classification of CwG as acute symptomatic seizures (ASS), aiming to facilitate its clinical management, particularly avoiding misdiagnosis of epilepsy and resultant overtreatment.

## 2 DEFINITION OF EPILEPSY AND ACUTE SYMPTOMATIC SEIZURES

An epileptic seizure is a transient occurrence of signs and/or symptoms due to abnormal, excessive, or synchronous neuronal activity in the brain. Moreover, epilepsy is a disorder of the brain which is defined by any of the following conditions: (1) at least two unprovoked (or reflex) seizures occurring >24 h apart; (2) one unprovoked (or reflex) seizure and a probability of further seizures similar to the general recurrence risk (at least 60%) after two unprovoked seizures, occurring over the next 10 years; and (3) diagnosis of an epilepsy syndrome.^16^ Epilepsy is characterized by an enduring predisposition to generate epileptic seizures and by the neurobiologic, cognitive, psychological, and social consequences of this condition.^17^ Thus, the epileptic seizure is completely different from epilepsy, both in the diagnostic criteria and associated treatment.

Acute symptomatic seizure was first proposed by the Commission on Classification and Terminology of ILAE in 1989 to facilitate epidemiological investigation of seizures.^18^ In 2010, ILAE defined ASS as an event, occurring in a close temporal relationship with an acute central nervous system (CNS) insult. In addition, the etiologies can be metabolic, toxic, structural, infectious, or due to inflammation.^19^ Even though the causes are various, all of them lead to the same final result: the excitability of the CNS was altered, leading to a transient lowering of the seizure threshold.^20^ There are two vital features of ASS, which are as follows: (1) there should always be an identifiable, concomitant acute and causal condition that has occurred close to the time of the seizure; and (2) ASS usually do not recur once the precipitating factor or condition has been removed or reversed, and the functional integrity of the CNS can be restored.^20^

In the acute stage of an infant's first seizure, even for experienced professionals, it is sometimes difficult to determine whether the child suffered the first seizure of epilepsy or an ASS. According to a previous study,^21^ 29.0/100,000 versus 42.5/100,000 of the children aged >2 months presenting with first seizure were classified subsequently as ASS and epileptic syndromes, respectively. In addition, Kikuchi K et al.^22^ retrospectively analyzed 41 children diagnosed with benign infantile seizures and 3 diagnosed with CwG in the first acute period of seizure; 26.8% (11/41) of the benign infantile seizures showed a “non‐benign" process over the follow‐up, which is defined as recurrent seizures without triggers or intellectual impairment. Importantly, there was no significance between CwG, “benign” and “non‐benign” groups regarding the demographic and clinical characteristics. Epilepsy tends to be provoked in some specific states such as fever, diarrhea, and other acute infection^23^; however, we should bear in mind that unprovoked seizures are always the core and essential item of epilepsy. Thus, the provoked factor is the main clue that contributes to distinguishing ASS from epilepsy, particularly for recurrent seizures within the same context as the first episode.

## 3 CwG FITS ALL THE CHARACTERISTICS OF ASS

It is generally agreed that seizures of CwG occur 1–6 days (an average of 2.3 days) after the onset of gastroenteritis typically,^24^, ^25^ and a few CwG occur 1–2 days before,^5^, ^26^ suggesting that seizures and acute gastroenteritis are essentially synchronized. To date, the pathogenesis of CwG remains unclear. While the CNS insult caused by an enterovirus, especially rotavirus, is considered as one of the pathogenesis of CwG. It has been reported that the positive rate of rotavirus in CwG ranges from 24.9% to 55.1%,^12^, ^27^, ^28^ and the rate of norovirus also gradually increased to 67.5% after the rotavirus vaccine was popularized.^28^, ^29^ Yet, we know less about the underlying pathogenesis of norovirus in CwG when compared with rotavirus. On one hand, rotavirus encodes the nonstructural protein 4 (NSP4).^30^ NSP4 has a direct membrane destabilization activity and causes endoplasmic reticulum membrane damage, which induces calcium homeostasis disruption and provokes seizure subsequently.^31^ On the other hand, increased levels of NO metabolites caused by rotavirus are involved in the ignition process of convulsion factors causing seizure.^32^, ^33^ In addition, the immaturity of the brain function may play a major role in the epileptogenesis in CwG since it frequently occurs in infants. On account of the immature neuronal dendrites, incomplete myelin sheaths, unbalanced brain chemistry, and neurotransmitters, infants may be more susceptible to seizure in response to mild stimuli.^14^, ^34^ Thus, it is reasonable to hypothesize that a lower seizure threshold may be triggered by intestinal infection or other factors in this entity. In conclusion, CwG is always provoked by causal acute gastroenteritis; it meets the first characteristic of ASS, namely, a concomitant and causal condition that has occurred close to the time of the seizure.

CwG has a favorable prognosis.^12^, ^27^, ^35^ First of all, CwG patients show normal neuropsychological development and even suffer a second episode of CwG.^9^, ^36^, ^37^ Secondly, the recurrent rate of afebrile seizure in CwG patients is about 7.7%, and 71% of them recurred with mild gastroenteritis,^12^ indicating that most of the seizure recurrences were still provoked. Besides, only a minority (0.04%–2.3%) of CwG developed epilepsy and responded well to levetiracetam or sodium valproate.^12^, ^38^ During the follow‐up, all of those epilepsy patients showed good drug response and the mean duration of therapy is 2 years.^35^, ^38^ In the end, although 21.9%–38.7% of the patients showed abnormal interictal EEG findings (encompassing slow background and/or spike‐wave discharge predominantly on the occipital, frontal‐parietal, and central area), most of them recovered normally during the follow‐up.^12^, ^26^, ^39^, ^40^, ^41^ Interestingly, some patients who were finally diagnosed with epilepsy had normal EEG at the first CwG period.^26^, ^38^ Likewise, another cohort study also stressed that abnormal EEG is not connected with the recurred seizure after CwG.^12^ In summary, the accumulating data suggested that the recurrence of CwG is rare in the absence of acute gastroenteritis, and the patients' neurocognitive developmental milestones is not affected by this entity. Thus, CwG accommodates the recoverability when the provoked factor disappears, which is the other criterion of ASS.

## 4 THE RISK FACTORS FOR THE RECURRENCE OF SEIZURES AFTER THE FIRST CwG

Although febrile seizure (FS), as another entity of ASS,^20^ differs from CwG in the definition (FS occurs with hyperpyrexia, whereas CwG is characterized by afebrile seizure), it shares many parallels with CwG. For instance, age of onset, provoked factors before a seizure, normal brain MRI, and cerebrospinal fluid examination as well as favorable prognosis.^42^ Both CwG and FS tend to recur. It is found that 22.7% of FS recurred after the initial episode.^43^ By contrast, CwG has a lower recurrence rate ranging from 4.3% to 20%.^26^, ^35^, ^38^, ^40^ Meanwhile, a study found that younger age, family history of FS, and low temperature before the initial seizure were powerful risk factors that determine the recurrence of febrile seizures.^44^ Similarly, another cohort study asserted that early age at onset (<18 months), repeated seizures over 24 h, and absence of fever during the CwG are the independent risk factors for the recurrence of afebrile seizure after CwG.^12^ What is more, Kaili Shi^13^ observed that a family history of seizures is a risk factor for CwG recurrence. It seems that the recurrence risk factors of FS and CwG overlap in a small age and family history. More intriguingly, few CwG suffered FS during follow‐up, suggesting FS and CwG can coexist in a single individual.^12^ Thus, CWG and FS may share the same pathogenesis, such as the immature brain.

It is well acknowledged that FS has a genetic predisposition.^45^ Comparatively, the data about CwG is very limited. A retrospective cohort research discovered the percentage of CwG patients with a family history in the recurrent was substantially higher than the nonrecurrent group's percentage (4.9% vs. 14.3%).^13^ In addition, Terrone^46^ reports two siblings who occurred to have CwG at the age of 20 months and 2 years, respectively, and both of them carry a novel pathogenic variant *SPTAN1*, both of which were seizure‐free over time. However, the daughter had normal cognitive functioning, while the son had developmental retardation. Meanwhile, other cohort studies^47^, ^48^ did not find a specific pathogenic gene of CwG, and the pathogenic genes and inherited pattern of CwG still need to be further explored.

## 5 EPILEPSY AFTER CwG

It has been reported that the risk for subsequent epilepsy in FS is 6.4%,^43^ while this proportion for CwG ranges from 0.5% to 2.3%.^12^, ^38^ In a cohort study of 25 CwG patients,^38^ only one patient eventually developed epilepsy. This patient had a family history of epilepsy and suffered recurrent focal seizures at the age of 3 years. The EEG showed explosive increased activity in the bilateral frontal regions. Finally, the patient was treated with sodium valproate, which was further discontinued during the follow‐up. In another cohort study in Italy,^26^ three patients (3/128) were diagnosed with epilepsy. One of them showed spikes and spike waves mainly in the temporoparietal and frontotemporal region during interictal EEG, and the remaining two were normal. The neuropsychological and motor development of the three patients was normal throughout the follow‐up period.

Epilepsy after CwG has many commonalities with self‐limited infantile epilepsy (SeLIE) proposed by the ILAE.^49^ On the one hand, they affect the same population: the infants and toddlers who are previously healthy and normally developed. The onset age of SeLIE is 3–20 months, with the peak at 6 months, and SeLIE usually has no history of convulsion during the neonatal period.^49^ A cohort study^12^ showed that the median age of the patients diagnosed with epilepsy after CwG was 7.5 months (IQR 6.7–14.7) in otherwise healthy children, which is close to the peak of SeLIE incidence. On the other hand, epilepsy after CwG and SeLIE have similar seizure characteristics^49^, ^50^, ^51^: a cluster of seizures, generalization after a focal origin seizure, short seizure duration less than 5 min usually, and without indicative changes on EEG. Most importantly, both of these two entities have the same favorable prognosis. Combined with epilepsy after CwG has good responses to anti‐seizure medications, we propose that epilepsy after CwG may be classified as SeLIE.

## 6 SUMMARY

**FIGURE 1:**
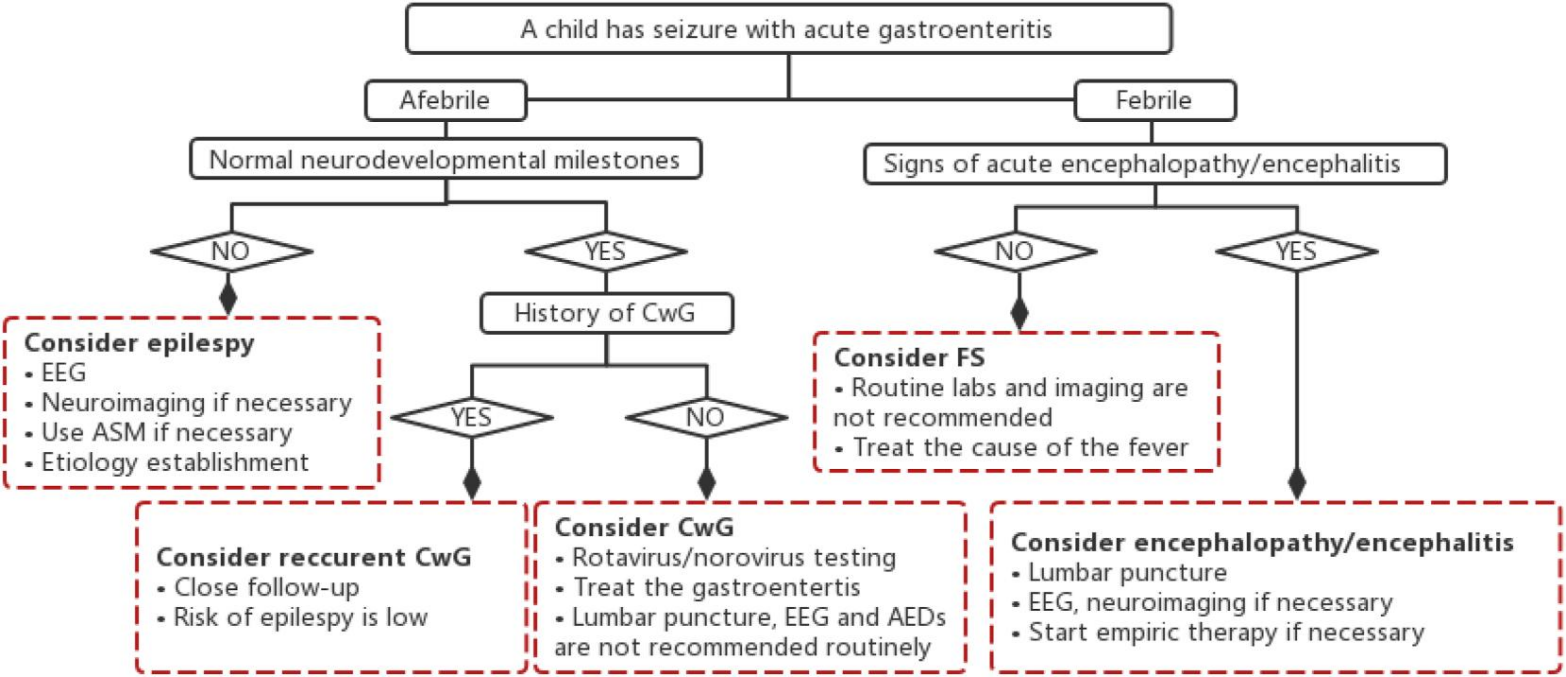
Assessment algorithm for an infant who has seizure with gastroenteritis. ASM, Anti‐seizure medicine; ASS, acute symptomatic seizure; CNS, central nervous system; EEG, Electroencephalogram; and FS, febrile seizure.

Collectively, CwG can be classified as an acute symptomatic seizure rather than epilepsy since it fits all the characteristics of ASS. Analogous to the common genetic basis among febrile seizures and associated epilepsy syndromes,^46^ the underlying pathogenesis of CwG may involve the genetic predisposition, even causative genetic variants, that need to be tested in more patients. Epilepsy after CwG exists but is rare, which needs to be further confirmed in long‐term follow‐up with large‐scale samples. Figure 1 presents an assessment algorithm for infants with seizures and gastroenteritis, aiming to facilitate the pediatricians to identify and manage this specific condition more effectively. Both CwG and subsequent SeLIE have a favorable prognosis, which suggests that overdiagnosis and overtreatment of epilepsy should be avoided in clinical practice.

## AUTHOR CONTRIBUTION

Conceptualization: Tingsong Li and Duan Wang; Investigation: Duan Wang; Visualization: Lijuan Peng; Writing – Original Draft Preparation: Duan Wang and Lijuan Peng; Writing – Review & Editing: Tingsong Li and Nong Xiao.

## CONFLICT OF INTEREST STATEMENT

The authors declare no conflict of interest.

## ETHICS STATEMENT

Not applicable.

## Data Availability

Data sharing is not applicable to this article as no datasets were generated or analyzed during the current study.
